# Supplementary material for: pH Dependence of Amyloid‐β Fibril Assembly Kinetics: Unravelling the Microscopic Molecular Processes
Source: Angew Chem Weinheim Bergstr Ger. 2022 Oct 27;134(48):e202210675. doi: 10.1002/ange.202210675 (PMC10947324; doi:10.1002/ange.202210675)
Supplement: Supplementary file 1 — Supporting Information [file ANGE-134-0-s001.pdf]

Angewandte Chemie
Eine Zeitschrift der Gesellschaft Deutscher Chemiker

Supporting Information

pH Dependence of Amyloid-β Fibril Assembly Kinetics: Unravelling the Microscopic Molecular Processes

*Y. Tian, J. H. Viles**

Supplemental Methods:

Aβ Peptides

Synthetic Aβ40 and Aβ42 were purchased from EZBiolab Inc in a lyophilized form. Aβ peptides were synthesized by F-moc (N- (9-fluorenyl)methoxycarbonyl) chemistry, and were purified with reverse-phase high performance liquid chromatography. Sequence was verified by mass spectrometry. Unless otherwise stated all other solvents and reagents were purchased from Sigma-Aldrich.

Monomeric Aβ by size-exclusion chromatography (SEC)

The lyophilized Aβ peptides were solubilized in ultra-high quality (UHQ) water to 0.7 mg/ml at pH 10 and left at 4°C for 2 h. Aβ solution was then centrifuged for 15 min at 20,000 g at 4°C, to remove high molecular weight aggregates. Monomeric Aβ was isolated using AKTA FPLC with a Superdex 75 10/300 GL column (GE Healthcare) at a flow rate of 0.5 $mL \cdot min^{-1}$. The column was pre-equilibrated with 5 mM sodium phosphate buffer (pH 6.0). The Aβ peptides concentration were determined using ε_{280}=1280$\cdot cm^{-1}$ mol^{-1}. Negative-stain TEM and thioflavin T fluorescence assay confirmed that SEC-purified Aβ peptides were seed-free. Monomeric samples were used directly after SEC.

Fibril growth Assay

The kinetics of amyloid fibril formation were monitored by a fibril-specific fluorescent dye, thioflavin T (ThT). Monomeric Aβ peptides (5 μM) and ThT (10 μM) were placed in a 96-well plate in NaCl (50 mM) and sodium phosphate (50 mM) buffer from pH 6.0 to pH 8.0. All fibril formation was performed under quiescent conditions. Experiments were recorded using a BMG-Omega FLUOstar microplate reader (BMG Labtech, Aylesbury, UK), with excitation and emission filters at 440 nm and 490 nm respectively.

In the seeded aggregation assay 10% fibrils, 0.5 μM monomer equivalent, were added. Aβ fibril seeds were obtained by incubating 5 μM Aβ peptides in NaCl (50 mM) and sodium phosphate (50 mM) buffer from pH 6.0 to pH 8.0 at 30 °C for 4 days. Samples also contained DMSO 0.5% (v/v). The formation of Aβ fibrils was confirmed by ThT fluorescent assay and TEM imaging.

Fitting fibril growth curves

The empirical kinetic values for $t_{1/2}$ and t_{lag} were extracted from the data by fitting the fibril growth curve to the equation below [14]

$$Y = (y_i + m_i x) + \frac{v_f + m_f x}{1 + e^{-(\frac{x - xo}{\tau})}}$$

Where Y is the ThT fluorescence intensity, x is the time and x_o is the time at which the ThT fluorescence has reached half maximal intensity referred to as $t_{1/2}$. The initial and final fluorescence signals, y_i and y_f, were used to determine the time at 10% and 90% ThT maximal for a measure of the growth time, t_{growth}.

The pK_a at the midpoint of the titration for t_{lag} and $t_{1/2}$ were obtained by curve-fitting to a recast Henderson-Hasselbalch equation.

$$y = (y_{max} + y_{min}10^{(pKa - pH)})/(1+10^{(pKa - pH)})$$

Analysis of Aβ aggregation kinetics

Global kinetic analysis of Aβ peptides aggregation were analyzed using the AmyloFit platform.[10] The integrated rate law which based on Michaelis-Menten-Like kinetics for Aβ aggregation traces is:

$$\frac{M}{M(\infty)} = 1 - \left(1 - \frac{M(0)}{M(\infty)}\right) e^{-k_\infty t} \times \left(\frac{B_- + C_+ e^{\kappa t}}{B_+ + C_+ e^{\kappa t}} \times \frac{B_+ + C_+}{B_- + C_+}\right)^{\frac{K_\infty^2}{\overline{\overline{k}}_\infty \kappa}}$$

where the additional coefficients are functions of κ and λ:

$$B_\pm = (k_\infty \pm \overline{k}_\infty)/2\kappa$$

$$C_\pm = \frac{k_+[P]_0}{\kappa} \pm \frac{k_+ M(0)}{2m(0)k_+} \pm \frac{\lambda^2}{2\kappa^2}$$

$$k_\infty = 2k_+ P(\infty)$$

$$\overline{k}_\infty = \sqrt{k_\infty^2 - 2C_+ C_- \kappa^2}$$

which are two combinations of the microscopic rate constants of:

$$\lambda = \sqrt{2k_+ k_n m(0)^{nc}}$$

$$\kappa = \sqrt{2m(0)k_+ \frac{m(0)^{n2}k_2}{1 + m(0)^{n2}/K_M}}$$

where $m(0)$ is the initial monomer concentration, $M(0)$ is initial fibril mass concentration, $M(\infty)$ is mass concentration of fibrils at equilibrium, $P(0)$ is the initial aggregate concentration and $P(\infty)$ is the aggregates concentration of at equilibrium. The microscopic rate constants k_n, k_2, k_+ are the rate constants for primary nucleation, secondary nucleation, and elongation respectively. The exponents n_c and n_2 are the reaction orders for primary and secondary nucleation respectively, and K_M is the saturation constant for secondary nucleation.

Using predetermined values for K_M; and initial k_+k_n and k_+k_2 values, the experimental macro kinetic traces were globally fitting to the integrated rate law over the range of pH's. The microscopic rate constants k_n; k_+; and k_2 values were fitted to the fibril growth curves at pH 6.0, the other kinetic traces at increasing pH's, were then fitted in three scenarios in which only one of the rate constants were permitted to vary, while the other two remain constant. This approach has been used to investigate how increasing concentrations of an inhibitor of fibril formation effect individual microrate constants.[15] In a similar way raising the pH (concentration of hydroxide ions) also inhibits fibril formation.

Transmission electron microscopy

Aβ fibril samples were generated with the same protocol for Aβ fibril growth assay but without ThT addition. 5 μL aliquot of sample were added onto glow discharged carbon-coated copper grids (Agar Scientific, Essex, UK) by the droplet method then blotted after 90 seconds and rinsed with ddH_20 at room temperature. Glow discharge was carried out using the Pelco EasiGlow glow discharge unit. to negatively stain the assemblies a 5 μL uranyl acetate (2 % w/v) was used, then blotted and rinsed after 10 seconds. Images were recorded by a JEOL JEM-1230 electron microscope (JEOL, Ltd., Japan) at 80,000 magnifications, operated at 120 kV, paired with a 2k Morada CCD camera and corresponding Olympus iTEM software package (Olympus Europa, UK). Node-to-node fibril distance was measured using image-J software.

	pK_a
t_{lag} (Aβ40)	7.07 +/- 0.05
$t_{1/2}$ (Aβ40)	7.01 +/- 0.02
$k_n(app)/k_n$ (Aβ40)	6.95 +/- 0.03
t_{lag} (Aβ42)	6.97 +/- 0.03
$t_{1/2}$ (Aβ42)	6.96 +/- 0.03
$k_n(app)/k_n$ (Aβ42)	7.09 +/- 0.03
N-terminus	7.9
His6; His13; His14	6.67; 6.64; 6.67

Table S1: Midpoint (pKa) of change in t_{lag}; $t_{1/2}$ and $k_n(app)/k_n$ for Aβ40 and Aβ42 with pH, compared with histidine and N-terminal pKa. Mean pKa of three histidine titrating plus the N-terminus is equal to 7.0, which is very close to the midpoint of the kinetic data.

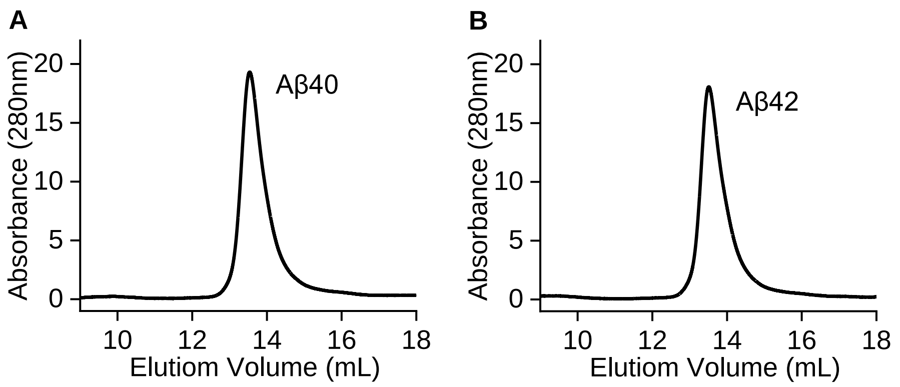

Figure S1. Isolation of Aβ40 (A) and Aβ42 (B) monomer by size exclusion chromatography.

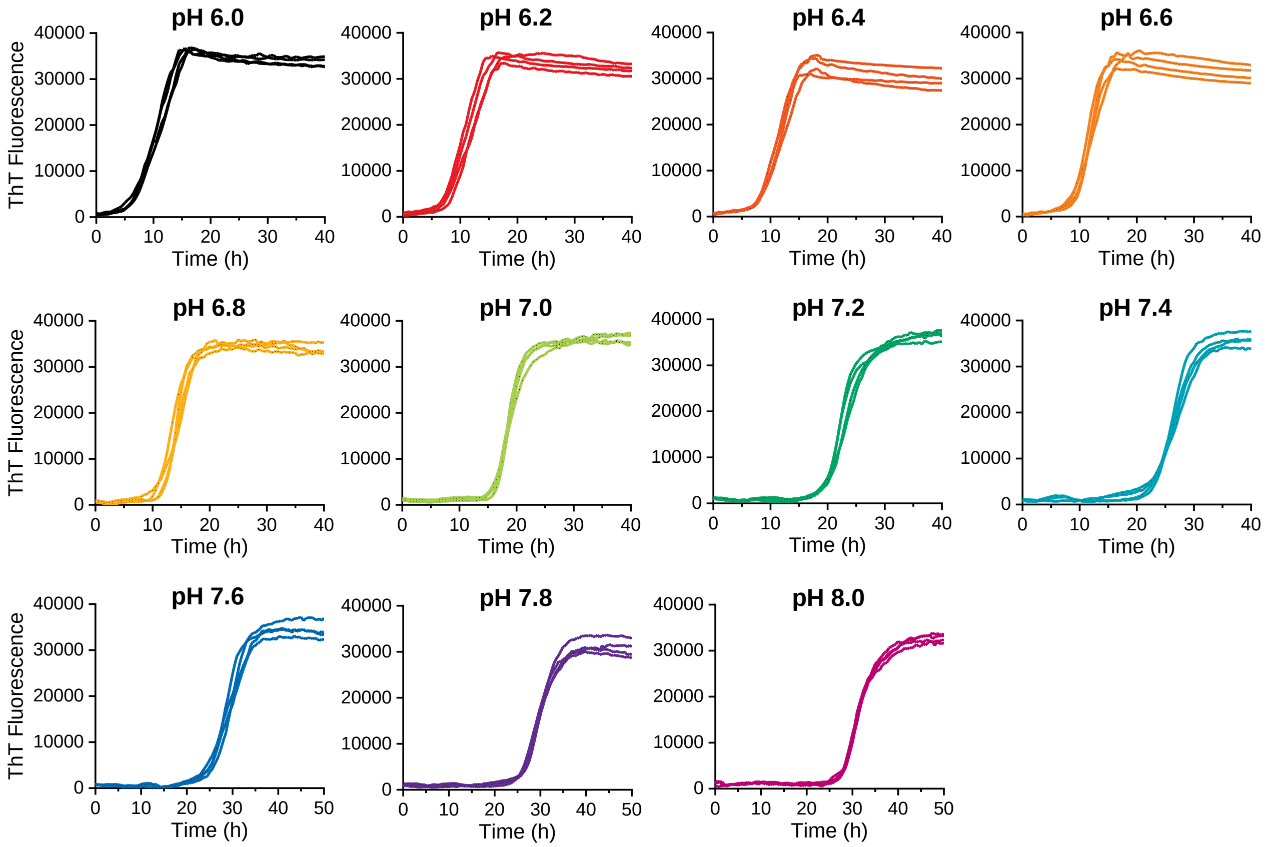

Figure S2. pH-dependent fibril formation kinetics of Aβ40. Plots of the kinetics for Aβ40 (5 μM) in 50 mM sodium phosphate and 50 mM NaCl buffer with ThT (10 μM) at 30°C under quiescent conditions, at pH 6.0, 6.2, 6.4, 6.6, 6.8, 7.0, 7.2, 7.4, 7.6 and 8.0. N= 4 traces for each condition.

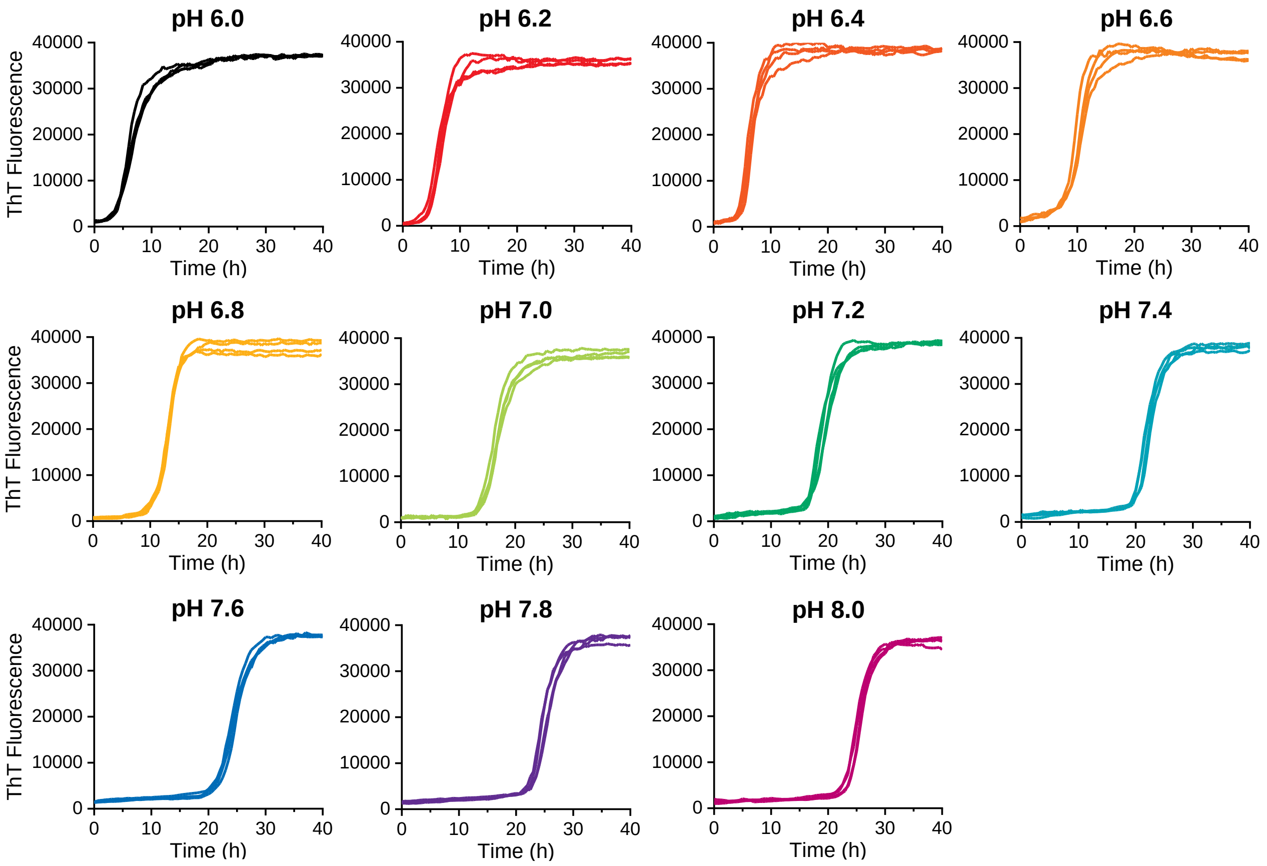

Figure S3. pH-dependent fibril formation kinetics of Aβ42. Plots of the kinetics for Aβ40 (5 μM) in 50 mM sodium phosphate and 50 mM NaCl buffer with ThT (10 μM) at 30°C under quiescent conditions, at pH 6.0, 6.2, 6.4, 6.6, 6.8, 7.0, 7.2, 7.4, 7.6 and 8.0. N= 4 traces for each condition.

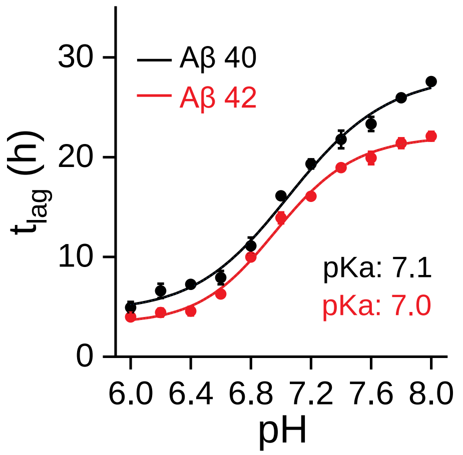

Figure S4. Plots of t_{lag} for Aβ40 and Aβ42 aggregation *versus* pH. Error bars represent standard errors of the mean (SEM) of four replicates.

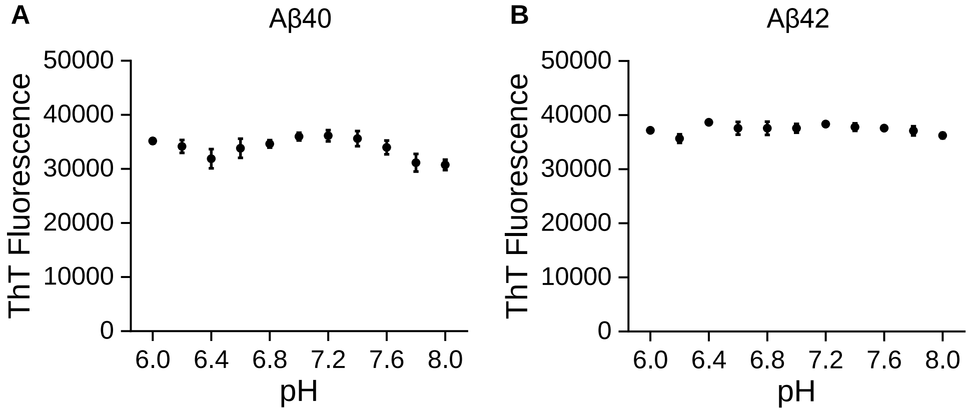

Figure S5. ThT fluorescence intensity *versus* pH. Aβ40 (A) and Aβ42 (B), derived from data in Figure S2 and S3.

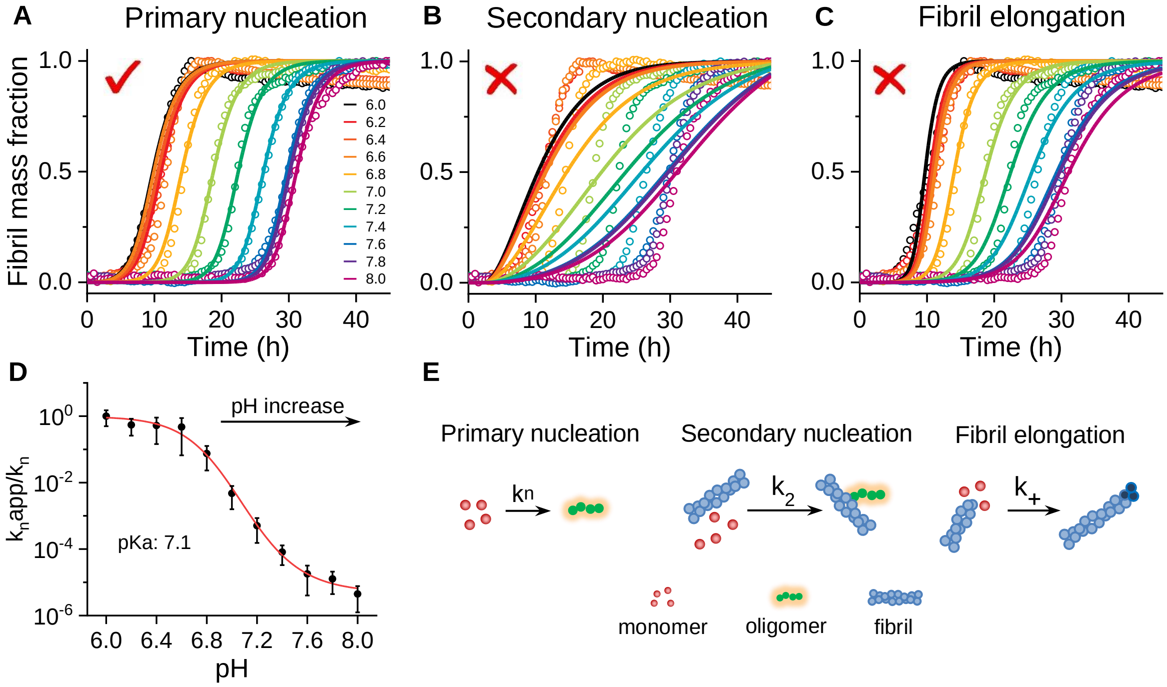

Figure S6. pH effects primary nucleation process of Aβ40 aggregate. (A-C) Kinetics profiles of Aβ40 (5 μM) at pH 6.0, 6.2, 6.4, 6.6, 6.8, 7.0, 7.2, 7.4, 7.6 7.8 and 8.0, from left (black, pH 6.0) to right (purple, pH 8.0). The solid lines represent global fits of the kinetic traces when only primary nucleation (A), secondary nucleation (B) and fibril elongation (C) rate constants are altered to fit pH dependent traces. (D) Change in primary nucleation rate constants (k_n) *versus* pH, derived from global fits in Figure 2A. (E) Schemes of the microscopic steps for primary nucleation, secondary nucleation and fibril elongation. Error bars represent standard errors of the mean (SEM) of four replicates.

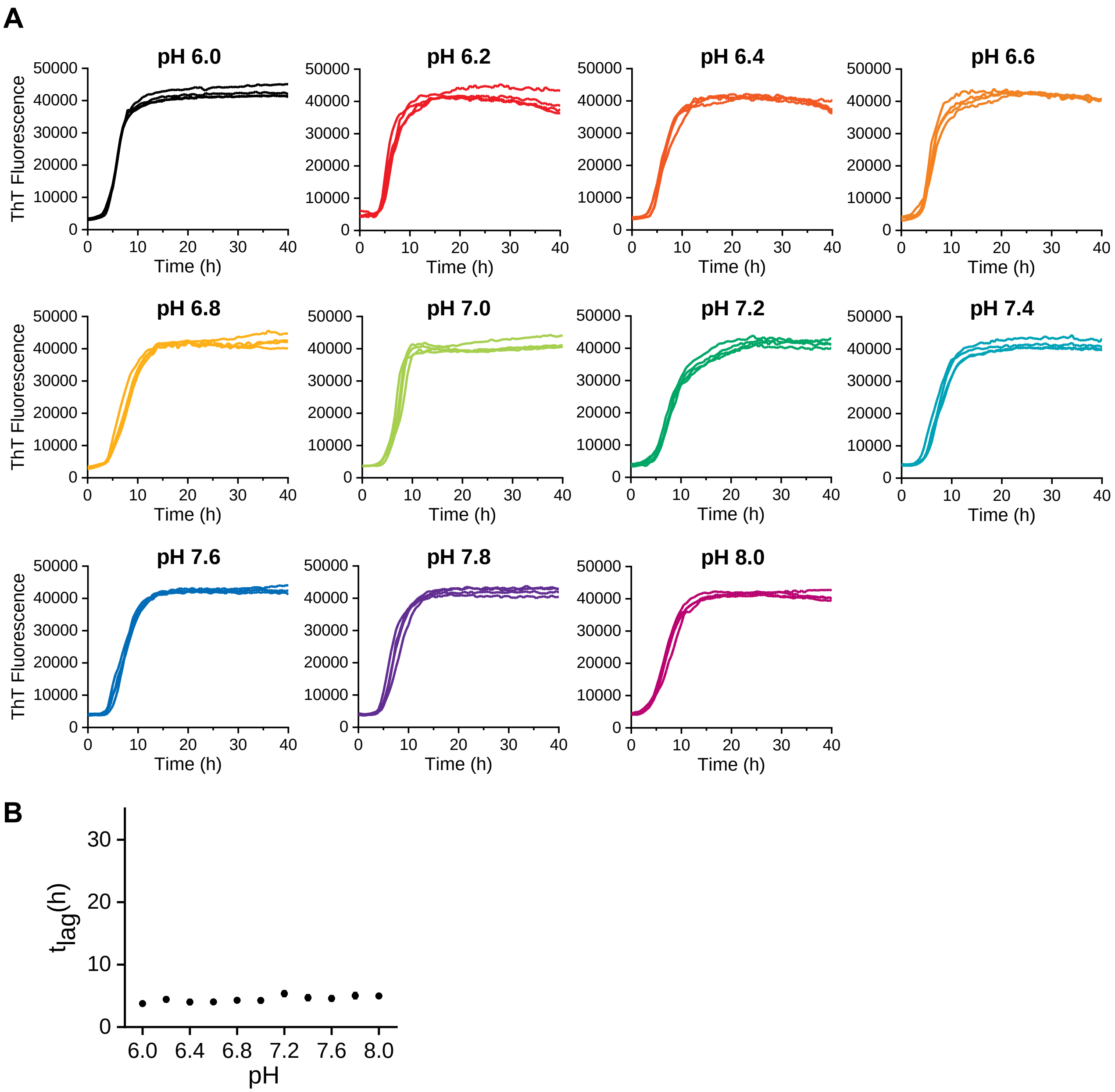

Figure S7. Seeded fibril formation is pH independent. (A) ThT kinetic traces (n=4) at pH 6.0, 6.2, 6.4, 6.6, 6.8, 7.0, 7.2, 7.4, 7.6 and 8.0. for Aβ40 (5 μM) with 10% fibril seed. 50 mM sodium phosphate and 50 mM NaCl buffer with 10 μM ThT at 30 °C under quiescent conditions. This seeded kinetics indicates secondary nucleation (k_2) and elongation (k_+) rates are independent of pH. **(B)** Plots of t_{lag} for Aβ40 with 10% seeds. Error bars represent standard errors of the mean (SEM) of four replicates.

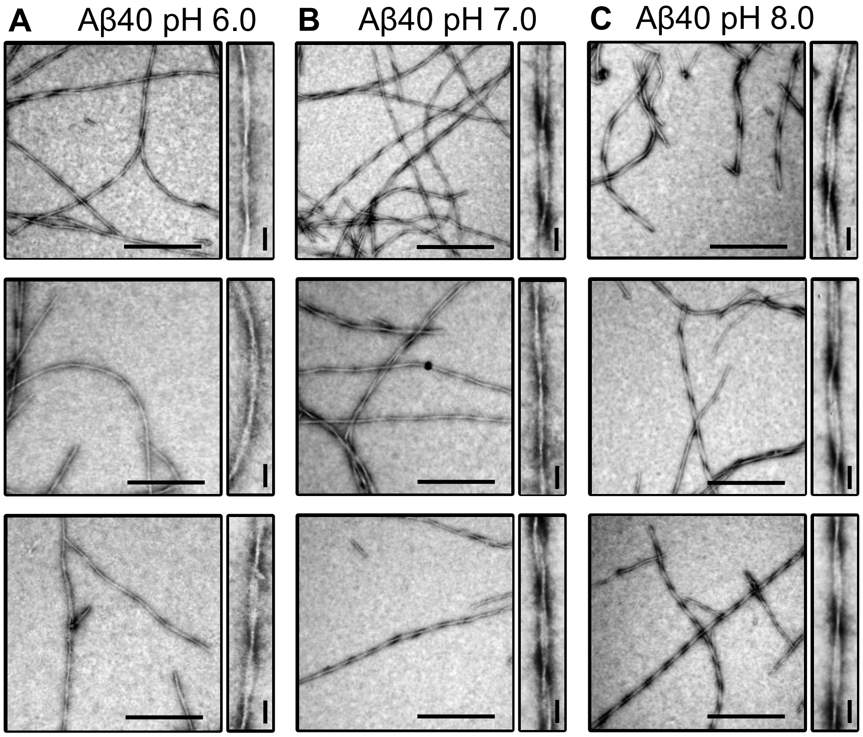

Figure S8. TEM fibril images produced at pH 6.0, 7.0 and 8.0 for Aβ40. Scale bars: 500 nm, right panel: 50 nm. pH has negligible effect on the morphology of Aβ40. Typical node-to-node twist periodicity 141 nm.

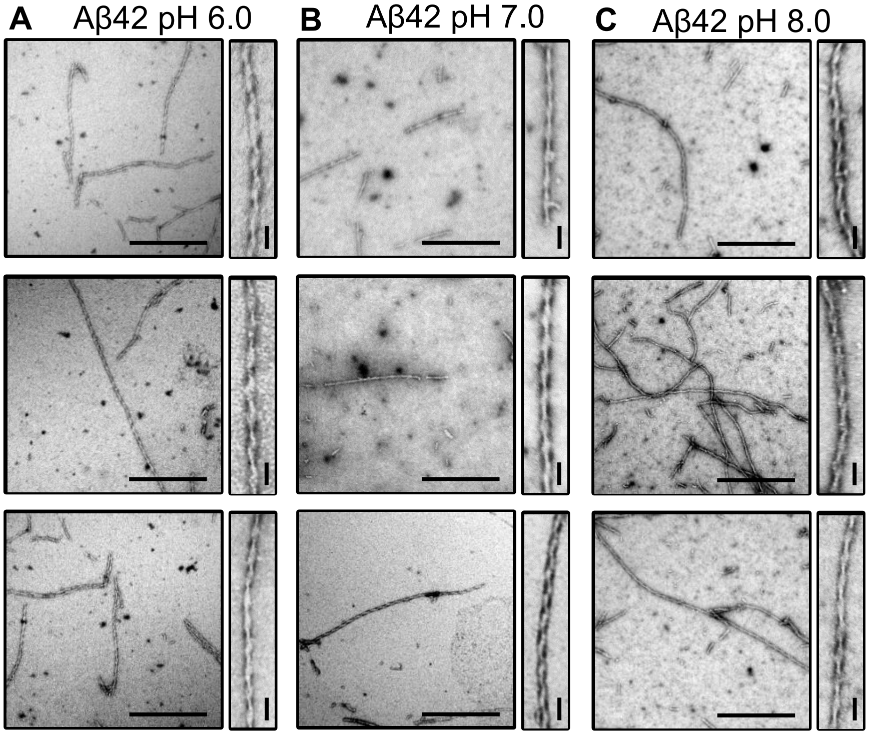

Figure S9. TEM fibril images produced at pH 6.0, 7.0 and 8.0 for Aβ42. Scale bars: 500 nm, right panel: 50 nm. pH has negligible effect on the morphology of Aβ42. Typical node-to-node twist periodicity 31 nm.

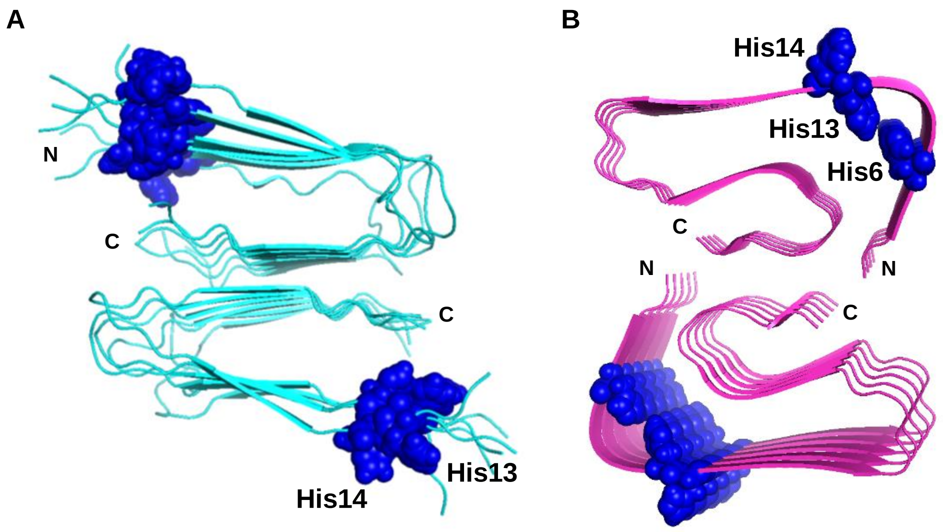

Figure S10: Structures of Aβ40 and Aβ42 fibrils. Position of titrating His6; His13; His14 residues, in blue. (A) Aβ40 showing ordered residues 9-40, PDB ID: 2LMO. His13 and His14 are on surface of fibril near disorder N-terminus. (B) Aβ42 all residues; PDB ID: 5OQV. Only His14 is on lateral surface of fibril in this Aβ42 structure. Note the different 'U' and 'S' shaped topologies for Aβ40 and Aβ42 respectively, a stack of 6 and 5 Aβ monomers are shown within the fibril. Most atomic resolution structures of Aβ42 fibrils indicate the N-terminal residues, up to residue 14, remain unstructured and not part of the fibril core structure PDB ID: 5KK3 and 2NAO, include all three histidine's.[19] Image generated with PyMOL.
